# Preoperative TACE with PD-1 inhibitors and TKIs in beyond “up-to-seven” hepatocellular carcinoma: a propensity score matched analysis

**DOI:** 10.3389/fimmu.2025.1657371

**Published:** 2025-11-26

**Authors:** Deyuan Zhong, Yuxin Liang, YaHui Chen, Yuhao Su, Qinyan Yang, Hongtao Yan, Xiaolun Huang, Ming Wang

**Affiliations:** Department of Liver Transplantation Center and Hepato-Biliary-Pancreatic (HBP) Surgery, Sichuan Clinical Research Center for Cancer, Sichuan Cancer Hospital & Institute, Sichuan Cancer Center, School of Medicine, University of Electronic Science and Technology of China, Chengdu, China

**Keywords:** neoadjuvant therapy, hepatocellular carcinoma (HCC), up-to-seven, propensity score matching (PSM), hepatectomy

## Abstract

**Background:**

HCC patients beyond the “up-to-seven” criteria face a high risk of recurrence, and optimal perioperative strategies warrant further investigation. This study evaluated the efficacy and safety of neoadjuvant TACE combined with PD-1 inhibitors and TKIs in this population.

**Methods:**

We retrospectively analyzed 126 HCC patients who underwent curative liver resection between January 2021 and June 2024. Of these, 73 patients received preoperative TACE with PD-1 inhibitors and TKIs (PST group), and 53 underwent direct surgery without preoperative therapy (Surgery group). PSM was applied to control for confounding factors, resulting in 39 matched pairs. The primary endpoints were RFS and OS, with secondary endpoints including intraoperative blood loss, surgical time, and complications.

**Results:**

After matching, the PST group demonstrated significantly superior RFS and OS compared to the Surgery group (P < 0.05). The median RFS was 26 months in the PST group versus 13 months in the Surgery group (P = 0.029). There were no significant differences in postoperative complications between the two groups (P = 0.365). The PST group exhibited longer surgical times (P < 0.001) and slightly higher intraoperative blood loss, suggesting increased surgical complexity due to preoperative therapy. Treatment-related adverse events were predominantly grade 1-2, with few grade 3–4 events.

**Conclusion:**

Preoperative TACE with PD-1 inhibitors and TKIs was associated with improved survival without increasing severe complications in beyond “up-to-seven” HCC, suggesting a promising perioperative strategy for high-risk patients.

## Background

Hepatocellular carcinoma (HCC) is the sixth most common malignancy worldwide and the third leading cause of cancer-related deaths (1). Despite advances in screening, liver function protection, and surgical techniques, the prognosis remains suboptimal. The five-year recurrence rate is as high as 70% (2). High-risk subgroups, particularly those with large tumor burden and multifocal distribution, face a significantly increased risk of postoperative recurrence (3, 4). This is a key factor limiting long-term survival.

The “up-to-seven” criteria, which combine the largest tumor diameter and the number of nodules, offer a concise and practical method for assessing tumor burden (5). This tool has been widely adopted for risk stratification in liver transplantation and liver resection patients (6). However, patients exceeding these criteria often demonstrate greater biological invasiveness and tumor heterogeneity. They have a limited response to traditional treatments and significantly poorer postoperative survival compared to those within the criteria (7, 8). For this high-risk population, effective perioperative management strategies remain scarce, highlighting the need for more synergistic treatment approaches.

Recently, neoadjuvant therapy has gained attention in the treatment of HCC (9, 10). Neoadjuvant therapy has proven effective in reducing tumor size and improving postoperative survival outcomes in various solid tumors (11). In HCC, its theoretical foundation is well-established. Primary tumors can continue to release antigens while intact, promoting tumor-specific T cell expansion and remodeling the immune microenvironment. This enhances the response to immune checkpoint inhibitors (ICIs) (12). Preliminary clinical studies have explored the potential of neoadjuvant immunotherapy and combination treatments in downstaging HCC and improving long-term prognosis (13–15). However, most of the available evidence remains exploratory, and there is a lack of systematic studies focusing on the high-risk beyond “up-to-seven” population.

The combination of transarterial chemoembolization (TACE), PD-1/PD-L1 inhibitors, and multi-target tyrosine kinase inhibitors (TKIs) has been extensively studied in advanced HCC (16, 17). This regimen not only exhibits direct antitumor effects, but also induces tumor antigen release, remodels the tumor immune microenvironment (TIME), promotes immune cell infiltration, and inhibits immune escape. These effects synergistically enhance antitumor activity (18–20). Early real-world studies have demonstrated higher objective response rates, prolonged progression-free survival (PFS), and a favorable safety profile in advanced HCC (21, 22). Given its potential for tumor shrinkage and systemic control, this triple regimen is anticipated to be an effective preoperative strategy for patients beyond the “up-to-seven” criteria. It can help achieve tumor downstaging, reduce postoperative recurrence risk, and prolong long-term survival.

There are currently no systematic studies examining the use of this triple neoadjuvant therapy in patients with beyond “up-to-seven” HCC. To address this gap, this study retrospectively analyzes the prognosis and safety outcomes between patients with beyond “up-to-seven” HCC who underwent successful liver resection after preoperative TACE combined with immunotherapy and targeted therapy, and those who underwent surgery alone. The aim is to provide evidence-based recommendations and practical references for perioperative management strategies in this high-risk population.

## Methods

### Study population and inclusion/exclusion criteria

From January 2021 to June 2024, we retrospectively analyzed 182 patients with HCC who were evaluated for liver resection at Sichuan Provincial People’s Hospital and Sichuan Cancer Hospital. Eligible patients were required to have an HCC diagnosis confirmed by both preoperative imaging and postoperative pathology, with a tumor burden exceeding the “up-to-seven” criteria (i.e., the sum of tumor number and maximum diameter >7). Surgical candidacy was determined by a multidisciplinary team (MDT). Only patients deemed technically resectable at the time of MDT evaluation were eligible. Among them, some proceeded directly to surgery, while others underwent elective surgery following a planned course of preoperative therapy.

In this study, such therapy was defined as neoadjuvant treatment—systemic therapy administered to patients already considered resectable, with the goal of improving surgical safety and long-term outcomes. In contrast, patients who required systemic therapy to achieve resectability (conversion therapy) were not enrolled. Importantly, only patients who ultimately underwent curative-intent (R0) resection were included in the analytic cohort. Patients who initiated but did not complete preoperative therapy due to disease progression, treatment-related adverse events, or personal choice did not undergo surgery and were therefore excluded. Inclusion criteria were as follows: (1) age 18–80 years, fit for surgery and general anesthesia; (2) initial diagnosis of HCC confirmed by imaging and pathology; (3) tumor burden beyond the “up-to-seven” criteria; (4) MDT-confirmed resectability, suitable for direct surgery or elective surgery after preoperative therapy; and (5) complete clinical, surgical, and follow-up data. Exclusion criteria included: (1) other active malignancies or distant metastases; (2) preoperative radiotherapy; (3) recurrent or secondary HCC; (4) severe liver dysfunction or other organ failure; and (5) failure to undergo liver resection.

**Figure 1:**
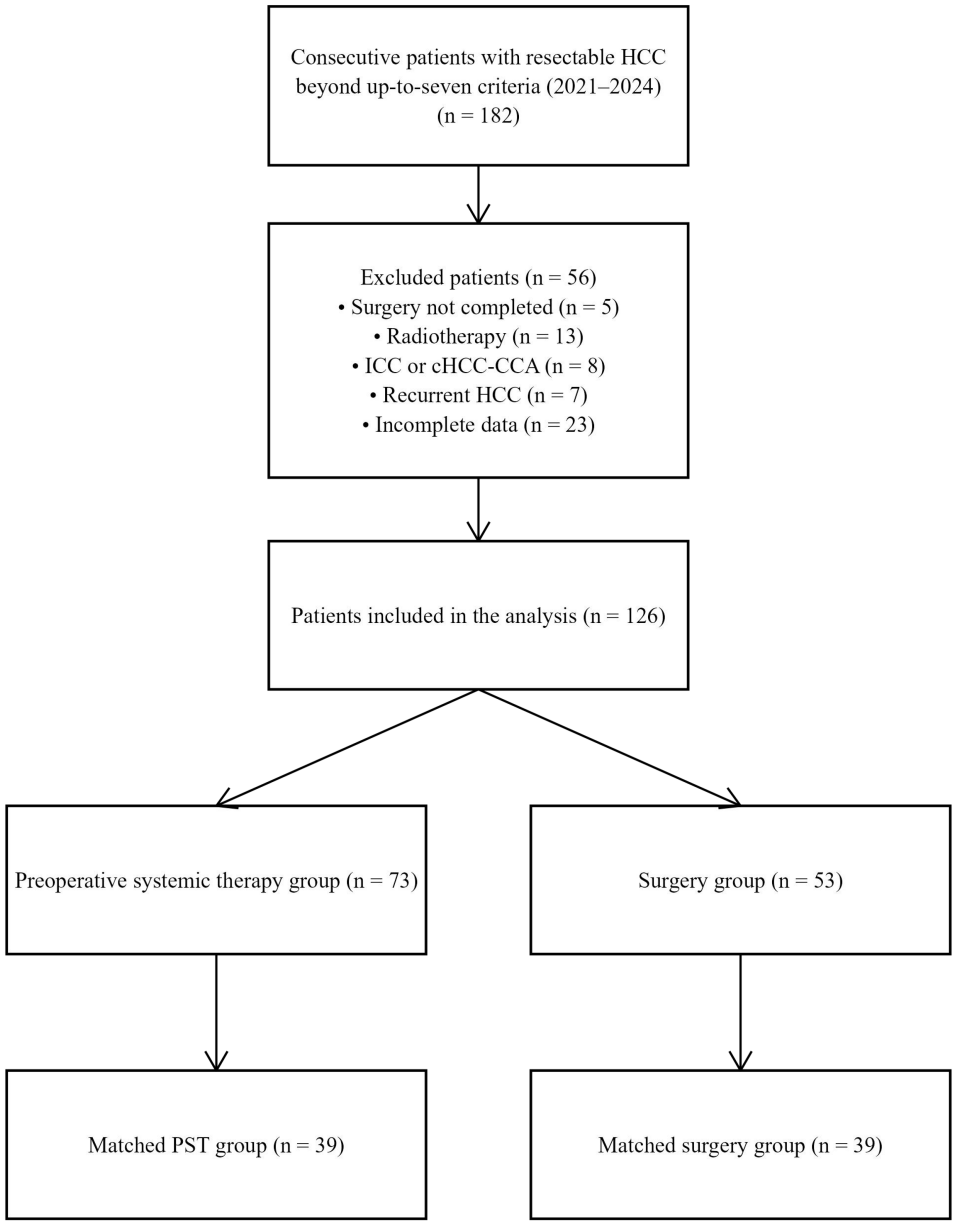
Flowchart of patient selection and grouping. HCC, hepatocellular carcinoma; ICC, intrahepatic cholangiocarcinoma; HCC-ICC, combined hepatocellular–cholangiocarcinoma; PST, preoperative systemic therapy; PSM, propensity score matching.

Ultimately, a total of 126 patients who underwent curative-intent resection for beyond “up-to-seven” HCC were included. Among them, 73 received preoperative combination therapy with TACE, PD-1 inhibitors, and TKIs (hereafter referred to as the PST group), while 53 underwent direct surgery without preoperative therapy (Surgery group). After 1:1 PSM, 39 matched pairs were obtained. The study flowchart is shown in **Figure 1**.

### Treatment regimen

Patients in the PST group received preoperative treatment consisting of TACE combined with PD-1 inhibitors and TKI. TACE was performed by experienced interventional radiologists using doxorubicin and oxaliplatin as chemotherapeutic agents, iodized oil, and embolic microspheres for arterial embolization to reduce tumor blood supply. TACE was repeated as needed, with an interval of 4–6 weeks, according to current guidelines (23). In this cohort, the median number of TACE sessions was 1 (range, 1–3). Systemic therapy consisted of lenvatinib (8 mg/day for body weight <60 kg or 12 mg/day for ≥60 kg), which was discontinued 7 days before surgery, and a PD-1 inhibitor—sintilimab (200 mg every 3 weeks) or tislelizumab (200 mg every 3 weeks)—initiated within 1 week after the first TACE and discontinued 4 weeks before surgery (24). Among PST patients post-PSM, 56% received sintilimab and 44% received tislelizumab; detailed regimen distribution is provided in **Supplementary Table S2**. Doses and schedules of ICI and TKI were adjusted for adverse events according to approved drug labels.

The duration of preoperative therapy was individualized by the MDT based on tumor progression characteristics, radiologic response, and the overall clinical status. Imaging reassessment was performed 4–6 weeks after the initial TACE. Patients proceeded to surgery approximately 4 weeks after the final treatment, with preoperative holds as specified above. Liver resections were performed by the same surgical team across both centers using anatomical or non-anatomical techniques via open or laparoscopic approaches, with the intent to achieve R0 resection.

### Propensity score matching

To minimize baseline differences and reduce confounding effects, 1:1 PSM was performed using a nearest-neighbor algorithm without replacement and a caliper width of 0.2 standard deviations of the logit of the propensity score. Propensity scores were derived from a logistic regression model including clinically relevant baseline covariates: age, sex, BCLC stage, tumor maximum diameter, number of nodules, Child–Pugh score, ALT, albumin, AFP, platelet count, and HBV infection status. All covariates were entered simultaneously into the model without tiered prioritization. Matching was not flexed beyond the specified caliper; patients without suitable matches were excluded from the matched cohort. Covariate balance was assessed using standardized mean differences (SMD), with SMD < 0.1 considered indicative of good balance.

### Outcome measures and follow-up

The primary endpoints were recurrence-free survival (RFS) and overall survival (OS). RFS was defined as the time from liver resection to the first documented recurrence (radiologically or pathologically). OS was defined as the time from surgery to death or last follow-up. Secondary endpoints included intraoperative blood loss, surgery time, postoperative complications (classified according to Clavien–Dindo), and adverse events related to preoperative systemic therapy (according to the Common Terminology Criteria for Adverse Events version 5.0). Follow-up was conducted through outpatient visits, telephone interviews, and imaging assessments, with the last follow-up date in March 2025. The follow-up endpoint for patients lost to follow-up was defined as the last valid follow-up date.

### Statistical analysis

Continuous variables were expressed as means ± standard deviation (SD) or medians (interquartile range), depending on distribution. Group comparisons were performed using Student’s t-test for normally distributed data or the Mann–Whitney U test for non-normally distributed data. Categorical variables were expressed as frequencies (percentages) and compared using Pearson’s chi-squared test or Fisher’s exact test. Survival curves were generated using the Kaplan–Meier method, with between-group differences assessed by the log-rank test. To further evaluate robustness, exploratory stratified analyses were performed in the post-PSM cohort according to microvascular invasion (MVI: present vs absent) and histologic differentiation (Edmondson–Steiner grade I–II vs III–IV). Within each stratum, survival outcomes between the PST and surgery groups were compared using Kaplan–Meier curves and log-rank tests. These analyses were exploratory in nature and were not adjusted for multiplicity. Cox proportional hazards models were then applied for univariate and multivariate analyses. To assess whether preoperative systemic treatment (PST) independently influenced postoperative outcomes, multivariable Cox models were constructed in an explanatory framework. Variables with P < 0.20 in univariate analysis, together with clinically relevant potential confounders, were entered to maintain model parsimony and minimize potential overfitting. Hazard ratios (HRs) with 95% confidence intervals (CIs) were reported. All statistical analyses were conducted using SPSS (version 25.0, IBM Corp., Armonk, NY, USA) and R (version 4.5.0, R Foundation for Statistical Computing, Vienna, Austria), with two-sided P < 0.05 considered statistically significant.

## Results

### Patient characteristics and neoadjuvant therapy outcomes

Between January 2021 and June 2024, we screened 182 HCC patients whose tumor burden exceeded the “up-to-seven” criteria and who were assessed by MDT for surgical eligibility. Five patients who received preoperative systemic therapy were excluded due to failure to complete surgery: 3 patients declined surgery after achieving partial remission, 1 lost surgical eligibility due to disease progression, and 1 discontinued treatment due to gastrointestinal bleeding. Ultimately, 126 patients who underwent curative liver resection for beyond “up-to-seven” HCC were included. Of these, 73 patients received preoperative TACE combined with PD-1 inhibitors and TKIs (PST group), and 53 patients underwent surgery alone (Surgery group). After 1:1 PSM, 39 matched pairs were obtained. The baseline characteristics of both groups before and after matching are presented in **Table 1**. Following matching, the two groups were well balanced in terms of age, sex, BCLC stage, HBV status, tumor diameter and number, liver function parameters, and AFP levels. The SMD plot showed a significant improvement in the balance of major baseline variables after matching, indicating good balance (**Supplementary Figure S1**). In the matched PST group, all patients received lenvatinib combined with PD-1 inhibitors (28 patients with sintilimab, 11 with tislelizumab) and TACE. The distribution of TACE treatment frequency was as follows: 17 patients received 1 session, 20 received 2 sessions, and 2 received 3 sessions. The median number of TACE sessions was 1. The duration of neoadjuvant treatment ranged from 6 to 20 weeks, with a median treatment time of 14 weeks.

**Table 1:** Baseline characteristics of patients in the preoperative systemic therapy (PST) and surgery groups before and after propensity score matching (PSM).

Characteristics	Before PSM			After PSM		
	Surgery (n=73)	PST (n=53)	*P*	Surgery (n=39)	PST (n=39)	*P*
Age, Mean (SD)	57.93 (11.11)	56.60 (10.47)	0.499	58.36 (11.02)	58.23 (9.77)	0.957
Female, n (%)	9 (16.9)	10 (13.7)	0.447	8 (20.5)	6 (15.4)	0.768
BMI, Mean (SD)	22.45 (3.00)	22.54 (1.76)	0.859	22.32 (2.79)	22.64 (1.60)	0.529
CTP score, Mean (SD)	5.12 (0.33)	5.17 (0.47)	0.515	5.13 (0.34)	5.18 (0.51)	0.601
BCLC, n (%)			0.794			0.937
A	11 (15.1)	6 (11.3)		5 (12.8)	4 (10.3)	
B	49 (67.1)	36 (67.9)		26 (66.7)	27 (69.2)	
C	13 (17.8)	11 (20.8)		8 (20.5)	8 (20.5)	
Tumor Diameter, Mean (SD)	7.39 (1.50)	7.76 (1.83)	0.218	7.62 (1.62)	7.55 (1.84)	0.866
Tumor Number, Mean (SD)	2.11 (0.86)	2.15 (0.84)	0.788	2.13 (0.92)	2.08 (0.74)	0.787
HBV, n (%)	58 (79.5)	41 (77.4)	0.95	32 (82.1)	32 (82.1)	1
Liver cirrhosis, n (%)	50 (68.5)	28 (52.8)	0.109	21 (53.8)	23 (59.0)	0.819
ALB, Mean (SD)	38.58 (2.73)	37.88 (3.15)	0.188	38.38 (3.09)	38.25 (3.05)	0.848
TBIL, Mean (SD)	15.60 (6.86)	18.16 (10.75)	0.107	16.82 (6.08)	18.66 (12.09)	0.398
PT, Mean (SD)	12.07 (1.12)	12.06 (1.04)	0.95	12.09 (1.15)	11.97 (0.94)	0.592
PLT, Mean (SD)	160.59 (69.82)	163.11 (57.24)	0.83	158.03 (63.58)	161.03 (54.40)	0.823
ALT, Mean (SD)	47.42 (33.64)	51.13 (41.88)	0.583	53.33 (42.38)	52.56 (47.81)	0.94
WBC, Mean (SD)	6.16 (1.88)	6.38 (1.93)	0.522	6.21 (1.99)	6.11 (1.88)	0.834
NEU, Mean (SD)	4.26 (1.65)	4.33 (1.72)	0.827	4.21 (1.76)	4.18 (1.72)	0.944
LYM, Mean (SD)	1.27 (0.33)	1.42 (0.43)	0.024	1.36 (0.31)	1.31 (0.39)	0.517
hsCRP, Mean (SD)	18.32 (27.92)	14.01 (17.75)	0.325	20.04 (31.91)	13.17 (17.05)	0.24
AFP, Mean (SD)	461.95 (789.45)	574.54 (978.40)	0.476	609.88 (1027.54)	523.39 (872.17)	0.69

Continuous variables are presented as mean ± standard deviation (SD), and categorical variables are presented as number (percentage). P-values were calculated using the Student’s t-test or Mann–Whitney U test for continuous variables and the chi-square test or Fisher’s exact test for categorical variables, as appropriate. PSM was performed using 1:1 nearest-neighbor matching with a caliper of 0.2 based on clinical covariates.

PST, preoperative systemic therapy; PSM, propensity score matching; BCLC, Barcelona Clinic Liver Cancer; HBV, hepatitis B virus; CTP, Child–Turcotte–Pugh; ALB, albumin; TBIL, total bilirubin; PT, prothrombin time; PLT, platelet count; ALT, alanine aminotransferase; WBC, white blood cell; NEU, neutrophil count; LYM, lymphocyte count; hsCRP, high-sensitivity C-reactive protein; AFP, alpha-fetoprotein.

### Survival analysis

**Figure 2:**
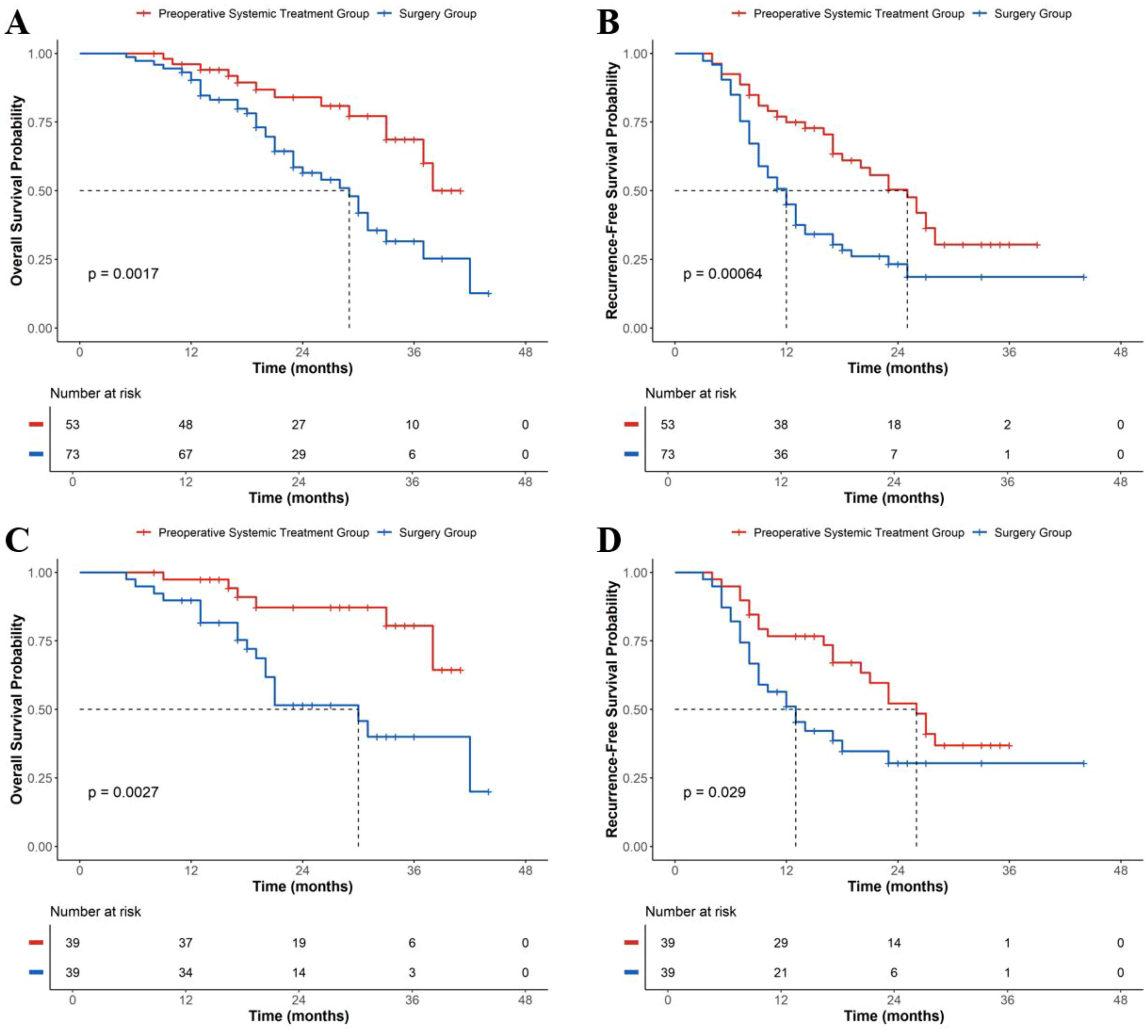
Kaplan–Meier analysis of overall survival (OS) and recurrence-free survival (RFS) between the preoperative systemic treatment (PST) group and surgery-alone group. **(A, B)** OS and RFS in the unmatched cohort. **(C, D)** OS and RFS in the matched cohort. Median survival times are indicated by dotted lines. Log-rank p-values are shown in each panel. PST, preoperative systemic treatment; OS, overall survival; RFS, recurrence-free survival; PSM, propensity score matching.

As of March 2025, the median follow-up time was 21 months (IQR: 15–32 months). Before matching, the median OS in the PST group was not reached, while the Surgery group had a median OS of 29 months, with a statistically significant difference (P = 0.0017, **Figure 2A**). The median RFS in the PST group was 25 months, significantly better than 12 months in the Surgery group (P = 0.00064, **Figure 2B**). After PSM, the median OS in the PST group remained unreached, while the Surgery group had a median OS of 30 months (P = 0.0027, **Figure 2C**). The median RFS in the PST group was 26 months, compared to 13 months in the Surgery group, with a statistically significant difference (P = 0.029, **Figure 2D**).

Exploratory stratified analyses further supported the robustness of the findings. In the MVI-positive subgroup, PST was associated with significantly better OS and RFS compared with surgery alone, while in the MVI-negative subgroup the median survival times remained longer in the PST group, although without statistical significance. Similarly, across both Edmondson–Steiner grade I–II and III–IV subgroups, PST demonstrated consistent directional survival advantages (**Supplementary Figures S2** and **S3**).

**Figure 3:**
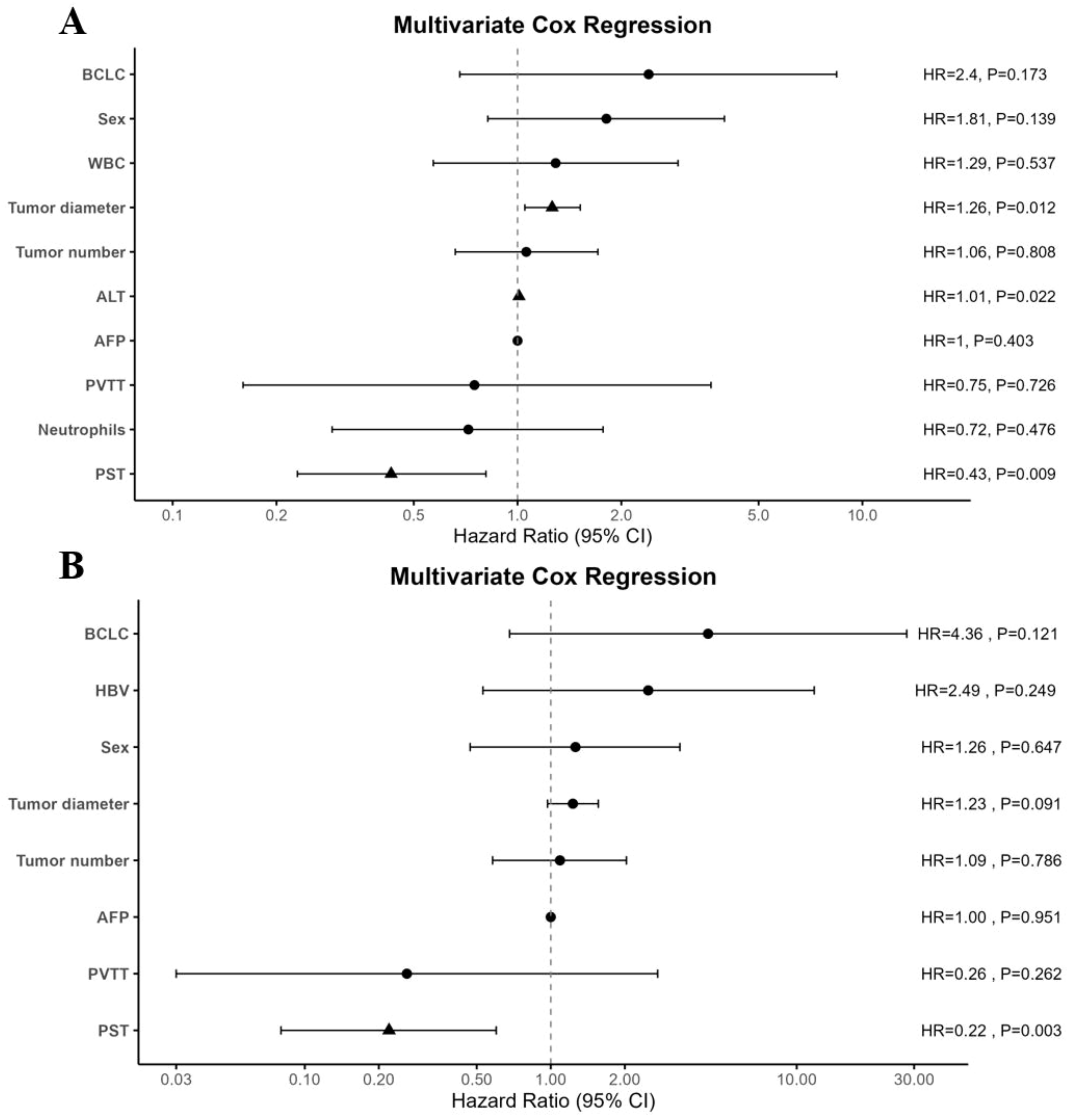
Forest plots of multivariate Cox regression analyses for recurrence-free survival (RFS) and overall survival (OS). **(A)** Multivariate Cox regression model for RFS in the propensity score–matched (PSM) cohort; **(B)** Multivariate Cox regression model for OS in the PSM cohort (n = 78). Hazard ratios (HRs), 95% confidence intervals (CIs), and p-values are shown for each variable. HR, hazard ratio; CI, confidence interval; PSM, propensity score matching; RFS, recurrence-free survival; OS, overall survival; PST, preoperative systemic treatment; PVTT, portal vein tumor thrombus; AFP, alpha-fetoprotein; ALT, alanine aminotransferase; HBV, hepatitis B virus; BCLC, Barcelona Clinic Liver Cancer.

Further Cox regression analysis is shown in **Table 2**. In univariate analysis, tumor diameter and receipt of preoperative systemic therapy were significantly associated with both RFS and OS (P < 0.05). In multivariate analysis, preoperative systemic therapy was an independent protective factor for RFS (HR = 0.427, 95% CI: 0.225–0.810, P = 0.009). Elevated ALT (HR = 1.008, 95% CI: 1.001–1.015, P = 0.022) and tumor diameter (HR = 1.263, 95% CI: 1.052–1.517, P = 0.012) were identified as independent adverse prognostic factors, suggesting that larger tumor burden is associated with higher recurrence risk. In the OS analysis, PST remained an independent protective factor (HR = 0.219, 95% CI: 0.079–0.602, P = 0.003) (**Figure 3**).

**Table 2:** Univariate and multivariate Cox regression analyses of factors associated with recurrence-free survival (RFS) and overall survival (OS).

Variables	RFS						OS					
	Univariate analysis			Multivariate analysis			Univariate analysis			Multivariate analysis		
	HR	95% CI	*P*	HR	95% CI	*P*	HR	95% CI	*P*	HR	95% CI	*P*
Age	1.009	0.976-1.043	0.612				1.019	0.975-1.065	0.401			
Sex	1.454	0.717-2.95	0.299	1.812	0.825-3.98	0.139	1.331	0.527-3.365	0.546	1.258	0.472-3.35	0.647
BMI	1.053	0.911-1.218	0.484				0.983	0.803-1.203	0.868			
CTP score	1.007	0.498-2.034	0.985				0.632	0.185-2.16	0.464			
BCLC	1.741	0.997-3.041	0.0514	2.3964	0.682-8.421	0.173	1.446	0.731-2.863	0.289	4.36	0.679-28.006	0.121
Tumor Diameter	1.226	1.033-1.454	0.0197	1.2633	1.052-1.517	0.012	1.279	1.044-1.566	0.0173	1.23	0.968-1.564	0.091
Tumor Number	1.044	0.698-1.561	0.834	1.061	0.657-1.713	0.808	1.263	0.786-2.03	0.334	1.09	0.584-2.035	0.786
HBV	1.225	0.547-2.745	0.622				2.949	0.678-12.825	0.149	2.494	0.528-11.783	0.249
Liver cirrhosis	1.436	0.784-2.632	0.241				1.432	0.609-3.369	0.411			
ALB	0.991	0.907-1.083	0.841				1.003	0.89-1.13	0.96			
TBIL	1.015	0.987-1.044	0.303				1	0.955-1.048	0.995			
PT	0.96	0.717-1.287	0.786				0.867	0.557-1.349	0.526			
PLT	1	0.995-1.004	0.878				0.998	0.991-1.004	0.477			
ALT	1.005	0.999-1.011	0.0885	1.008	1.001-1.015	0.022	1.005	0.996-1.014	0.256			
WBC	0.902	0.775-1.051	0.186	1.292	0.572-2.917	0.537	0.903	0.731-1.116	0.345			
NEU	0.881	0.741-1.048	0.152	0.72	0.292-1.775	0.476	0.892	0.7-1.137	0.358			
LYM	1.343	0.594-3.035	0.479				1.295	0.415-4.04	0.656			
hsCRP	0.999	0.986-1.011	0.843				0.996	0.975-1.017	0.684			
AFP	1	1-1	0.611	1	1-1	0.402	1	1-1.001	0.227	1	0.999-1.001	0.951
PVTT	1.759	0.888-3.483	0.105	0.7549	0.157-3.636	0.726	1.075	0.425-2.722	0.878	0.2622	0.025-2.716	0.262
Intraoperative blood loss	1.001	0.999-1.003	0.369				1.001	0.998-1.003	0.715			
Operation time	1.002	0.995-1.009	0.577				1.001	0.992-1.011	0.804			
Blood transfusion	1.65	0.695-3.914	0.256				1.016	0.301-3.43	0.979			
Tumor differentiation	1.196	0.634-2.254	0.581				1.704	0.678-4.279	0.257			
Clavien–Dindo grade	1.408	0.82-2.418	0.215				1.21	0.59-2.483	0.603			
PST	0.52	0.285-0.948	0.0327	0.4272	0.225-0.81	0.009	0.263	0.104-0.668	0.005	0.219	0.079-0.602	0.003

Hazard ratios (HRs), 95% confidence intervals (CIs), and P values are presented for each variable. Variables with P < 0.20 in the univariate analysis, as well as clinically relevant variables based on previous studies, were included in the multivariate model. Significant predictors (P < 0.05) in multivariate models are bolded.

RFS, recurrence-free survival; OS, overall survival; HR, hazard ratio; CI, confidence interval; BCLC, Barcelona Clinic Liver Cancer; HBV, hepatitis B virus; CTP, Child–Turcotte–Pugh; ALB, albumin; TBIL, total bilirubin; PT, prothrombin time; PLT, platelet count; ALT, alanine aminotransferase; WBC, white blood cell count; NEU, neutrophil count; LYM, lymphocyte count; hsCRP, high-sensitivity C-reactive protein; AFP, alpha-fetoprotein; PVTT, portal vein tumor thrombus; PST, preoperative systemic therapy.

### Surgical outcomes

Intraoperative blood loss in the PST group was slightly higher than in the Surgery group (420.5 ± 138.0 ml vs. 369.2 ± 130.1 ml), though the difference was not statistically significant (P = 0.095, **Table 3**). The surgical time in the PST group was significantly longer than in the Surgery group (210.8 ± 42.7 min vs. 178.5 ± 33.7 min, P < 0.001, **Table 3**). The proportion of intraoperative blood transfusions was similar between the two groups, indicating no significant difference in transfusion requirements.

**Table 3:** Comparison of intraoperative variables and postoperative complications between the PST and surgery groups.

Variables	PST (n=39)	Surgery (n=39)	P
Intraoperative blood loss, ml, mean (SD)	420.5 ± 138.0	369.2 ± 130.1	0.095
Operation time, min, mean (SD)	210.8 ± 42.7	178.5 ± 33.7	<0.001
Blood transfusion, n(%)	4 (10.3)	4 (10.3)	1
Postoperative complications, n(%)	18 (46.2)	22 (56.4)	0.365
Biliary leakage	2	4	
Bleeding	2	3	
Hepatic insufficiency	1	1	
Pleural effusion	3	4	
Peritoneal effusion	3	1	
Pulmonary infection	1	2	
Wound infection	2	3	
Clavien-Dindo Grade			0.144
I	28	22	
II	11	16	
III	0	1	
IV	0	0	

Surgical characteristics and postoperative complications were compared between the preoperative systemic therapy (PST) group and the surgery-alone group. Continuous variables, including intraoperative blood loss and operation time, are presented as mean ± standard deviation (SD) and were compared using independent samples t-tests. Categorical variables such as blood transfusion and overall postoperative complications are reported as number (percentage) and analyzed with chi-square or Fisher’s exact test as appropriate. The incidence of specific complications—including biliary leakage, bleeding, hepatic insufficiency, pleural effusion, peritoneal effusion, pulmonary infection, and wound infection—is summarized descriptively. The severity of postoperative complications was graded according to the Clavien–Dindo classification and compared using the Mann–Whitney U test.

PST, preoperative systemic therapy; SD, standard deviation.

The overall complication rate within 30 days after surgery did not significantly differ between the two groups (PST group 46.2%, Surgery group 56.4%, P = 0.365, **Table 3**). Common complications included bile leakage, postoperative bleeding, liver dysfunction, pleural and abdominal effusion, lung infection, and wound infection, with similar types and frequencies between the groups. Clavien–Dindo grade ≥ III severe complications were observed in only 1 case in the Surgery group, with no significant difference between the groups (P = 0.144), indicating that PST treatment did not significantly increase the risk of severe postoperative complications.

### Adverse events for neoadjuvant therapy

In the PST group, several treatment-related adverse events (TRAEs) were observed, most of which were grade 1–2, with relatively few grade 3–4 events (**Supplementary Table S1**). The most common adverse reactions included elevated transaminases (49.1%), fever (37.7%), abdominal pain (35.8%), and hypertension (24.5%). Three patients (5.7%) experienced grade 3 transaminase elevation, two (3.8%) had grade 3 hypertension, and one (1.9%) had grade 3 abdominal pain and thrombocytopenia.

No treatment-related deaths or grade 4 immune-related serious adverse events (such as immune hepatitis or pneumonia) were observed. Other adverse reactions such as diarrhea (7.5%), sensory disturbances (7.5%), rash (7.5%), thrombocytopenia (11.3%), and hyperbilirubinemia (9.4%) were mostly mild to moderate. Hypothyroidism was observed in 2 patients (3.8%), both grade 1. Increased creatinine and vomiting were observed in 2 patients (3.8%) each, with no severe toxicities reported. Overall, the PST regimen was well tolerated, with most patients successfully completing the neoadjuvant treatment.

## Discussion

This study, based on real-world data, evaluated the postoperative efficacy and safety of preoperative systemic therapy in HCC patients exceeding the “up-to-seven” criteria. After controlling for confounding factors using PSM, the PST group showed significantly better RFS and OS compared to the surgery-only group, with no increase in perioperative complication risk. These findings suggest that preoperative systemic therapy holds potential clinical value in high-risk, beyond “up-to-seven” HCC populations. After matching, both groups were well balanced in terms of baseline characteristics, such as age, sex, BCLC stage, tumor burden, and liver function, effectively reducing confounding bias. The PST group demonstrated superior survival outcomes, further supporting the use of neoadjuvant therapy in HCC patients with high tumor burden and high postoperative recurrence risk. Multivariate analysis identified preoperative systemic therapy as an independent protective factor for both RFS and OS, while tumor diameter and elevated ALT levels were independent adverse prognostic factors. This suggests that both tumor size and potential liver inflammation may jointly impact treatment efficacy. Exploratory subgroup analyses further support the robustness of our findings. In the post-PSM cohort, the survival advantage of PST was preserved across subgroups defined by MVI and histologic differentiation. The benefit reached statistical significance in MVI-positive patients, whereas in MVI-negative patients the differences did not achieve significance but remained directionally consistent, likely reflecting limited sample size and event numbers. Similarly, consistent benefits of PST were observed across differentiation subgroups, supporting the applicability of this strategy irrespective of tumor grade. Together, these results strengthen confidence in the stability of our primary outcomes and provide a solid foundation for comparison with prior neoadjuvant experiences in HCC and other solid tumors.

These findings are consistent with the growing trend of applying neoadjuvant therapy across solid tumors. Prior studies have shown that neoadjuvant therapies significantly improve pathological response and RFS in lung cancer, as well as PFS in melanoma (25, 26). In HCC, preoperative ICIs also show promise. Research indicates that 32% of HCC patients receiving neoadjuvant ICIs achieve major pathological response (MPR), and 18% achieve pathological complete response (pCR), significantly extending postoperative RFS (27). Building on these findings, our study advances the application of systemic therapy to the preoperative phase, focusing on the high-recurrence-risk beyond “up-to-seven” subgroup, providing further evidence for this approach. Notably, the IMbrave050 study’s subgroup analysis showed that, even in patients whose tumor burden exceeded the “up-to-seven” criteria, adjuvant therapy with atezolizumab and bevacizumab resulted in a trend of improved RFS. This further supports the potential value of immune combination strategies in high-risk HCC patients (28), and raises the question of the biological mechanisms underlying these benefits.

From a mechanistic perspective, the preoperative triple treatment regimen offers multiple synergistic anti-tumor effects (29). In this study, TACE was combined with systemic agents (TKIs and PD-1 inhibitors) as an integrated neoadjuvant regimen. Accordingly, the mechanistic rationale and clinical implications should be understood in the context of this combined loco-regional plus systemic approach, rather than TACE in isolation. TACE induces local hypoxia by blocking tumor blood supply, which activates HIF-1α and upregulates pro-angiogenic factors such as VEGF and PDGF. This can promote tumor angiogenesis (29, 30). Simultaneously, chemotherapy agents released during TACE induce tumor cell necrosis, antigen release, and inflammatory factor expression, enhancing tumor immunogenicity and converting “cold tumors” into “hot tumors,” thus improving immune therapy efficacy (18, 31, 32). TKIs block VEGF/PDGF signaling pathways, providing both anti-angiogenic and direct anti-cancer effects. These counteract the pro-angiogenic effects induced by TACE and enhance immune sensitivity by improving the tumor microenvironment (33, 34). However, TKIs may also exacerbate tumor hypoxia, leading to upregulation of PD-L1 expression, which may activate immune suppression and promote immune escape (35, 36). In this context, PD-1/PD-L1 inhibitors can relieve immune suppression, restore T-cell function, and reestablish tumor immune surveillance (37, 38). Preclinical and translational studies further suggest that TKIs augment PD-1 activity by improving antigen presentation and limiting suppressive cell infiltration (34). Importantly, systemic immune activation during the preoperative phase may induce immune memory before postoperative tolerance develops, enabling long-term surveillance of residual disease (12). In summary, this neoadjuvant triple regimen offers complementary and synergistic mechanisms that reduce tumor burden, optimize resection conditions, and potentially prolong long-term survival. Beyond HCC, the rationale of combining locoregional and systemic strategies is supported by evidence from other malignancies. Biomarker-driven interventions have been shown to reshape treatment outcomes in esophageal cancer, underscoring the importance of tailoring systemic therapy to biological context (39). Likewise, targeting both cancer cells and the tumor microenvironment represents a therapeutic principle highly relevant to our approach (40). Broader oncologic reviews have emphasized the historical evolution and future promise of combination therapy, situating our findings within this larger treatment paradigm (41). In addition, biomarker discovery is increasingly informing drug development and patient stratification (42), while the challenge of T cell exhaustion remains central to immunotherapy resistance. Recent studies identifying novel exhaustion markers in CD8^+^ T cells provide important mechanistic insight and further rationale for PD-1 based strategies in HCC (43).

In terms of safety, the PST group experienced significantly longer surgical times and a trend toward higher intraoperative blood loss compared with the Surgery group. These findings suggest that preoperative therapy may increase surgical complexity, potentially due to treatment-induced liver fibrosis, inflammation, or altered vascular supply. Importantly, however, this increased technical difficulty did not translate into higher perioperative risk. The overall complication rate within 30 days was comparable between groups, and severe complications (Clavien–Dindo grade ≥ III) occurred in only one patient in the Surgery group. These results indicate that, while resections after PST may be more demanding for the surgical team, the procedure remains safe in experienced centers. Most adverse events related to the neoadjuvant therapy were grade 1–2, with few grade 3–4 events, and the majority of patients successfully completed the planned treatment. Taken together, these findings support the feasibility and tolerability of the PST regimen. Future studies incorporating pathological and imaging correlates will be valuable to further elucidate the mechanisms underlying these operative changes and to optimize perioperative management.

This study has several limitations. First, the analytic cohort was restricted to patients who ultimately underwent curative-intent resection. Patients who failed to proceed to surgery were not included, as they lay outside the scope of this comparative analysis. This design ensured comparability with the direct surgery group and is consistent with previous large retrospective studies (44). Nevertheless, such exclusion inevitably introduces selection bias, as it preferentially retains patients with more favorable tumor biology and treatment tolerance. Consequently, the survival benefit of PST observed in this study may be overestimated, and the survival curves may appear more optimistic than would be expected in an intention-to-treat population. Second, the retrospective design and relatively small sample size limit the strength of the conclusions, as residual confounding from unmeasured variables (e.g., performance status, comorbidities, socioeconomic factors) cannot be fully excluded despite propensity score matching. In addition, the limited number of survival events—particularly for OS—raises the risk of overfitting in the multivariate Cox regression models, and these results should therefore be interpreted with caution. Third, treatment heterogeneity existed within the PST group in terms of both the specific PD-1 inhibitors and the number of TACE sessions. We reported the regimen distribution descriptively, but did not attempt to compare outcomes across regimens due to limited subgroup sizes. This heterogeneity reflects real-world MDT practice, yet it may affect the interpretation of results. Finally, mechanistic insight was constrained by the absence of biomarker correlates such as immune infiltration, PD-L1 status, or cfDNA kinetics, since biospecimens were not adequately preserved for this retrospective cohort. These limitations highlight the need for prospective, multicenter trials with standardized treatment protocols and integrated biomarker analyses to confirm the true clinical value and biological underpinnings of this strategy.

In conclusion, this study suggests that preoperative TACE combined with PD-1 inhibitors and TKIs is associated with improved survival and acceptable safety in patients with beyond “up-to-seven” HCC. This combined strategy may represent a promising perioperative option for high-risk patients and warrants validation in prospective studies to confirm its clinical value.

## Data Availability

The raw data supporting the conclusions of this article will be made available by the authors, without undue reservation.
