# Supplementary material for: Preoperative TACE with PD-1 inhibitors and TKIs in beyond “up-to-seven” hepatocellular carcinoma: a propensity score matched analysis
Source: Front Immunol. 2025 Nov 26;16:1657371. doi: 10.3389/fimmu.2025.1657371 (PMC12689949; doi:10.3389/fimmu.2025.1657371)
Supplement: Supplementary file 1 [file Table1.docx]

| Events, n(%) | PST (n=53) | |
| --- | --- | --- |
|  | Any Grade | Grade 3/4 |
| Abdominal pain | 19 (35.8) | 1 (1.9) |
| Fever | 20 (37.7) | 0 |
| Diarrhea | 4 (7.5) | 0 |
| Transaminitis | 26 (49.1) | 3 (5.7) |
| Hyperbilirubinemia | 5 (9.4) | 0 |
| Hypoalbuminemia | 3 (5.7) | 0 |
| Thrombocytopenia | 6 (11.3) | 1 (1.9) |
| Hypothyroidism | 2 (3.8) | 0 |
| Elevated creatinine | 2 (3.8) | 0 |
| Vomiting | 2 (3.8) | 0 |
| Paresthesia | 4 (7.5) | 0 |
| Rash | 4 (7.5) | 0 |
| Hypertension | 13 (24.5) | 2 (3.8) |

**Table S1. Treatment-related adverse events (TRAEs) during preoperative systemic therapy (PST; pre-PSM, n=53)**

Adverse events occurring during preoperative systemic therapy (n = 53) are summarized according to the Common Terminology Criteria for Adverse Events (CTCAE). Events are presented as number (percentage) by maximum severity, classified into any grade and grade 3/4.

**Abbreviations:** PST, preoperative systemic therapy; TRAEs, treatment-related adverse events; CTCAE, Common Terminology Criteria for Adverse Events.

| Characteristics | n (%) |
| --- | --- |
| Type of PD-1 inhibitors |  |
| Sintilimab | 22 (56%) |
| Tislelizumab | 17 (44%) |
| Number of TACE sessions |  |
| 1 | 29 (74.4%) |
| 2 | 8 (20.5%) |
| 3 | 2 (5.1%) |

**Table S2. Treatment characteristics in the PST group (post-PSM, n=39)**

**
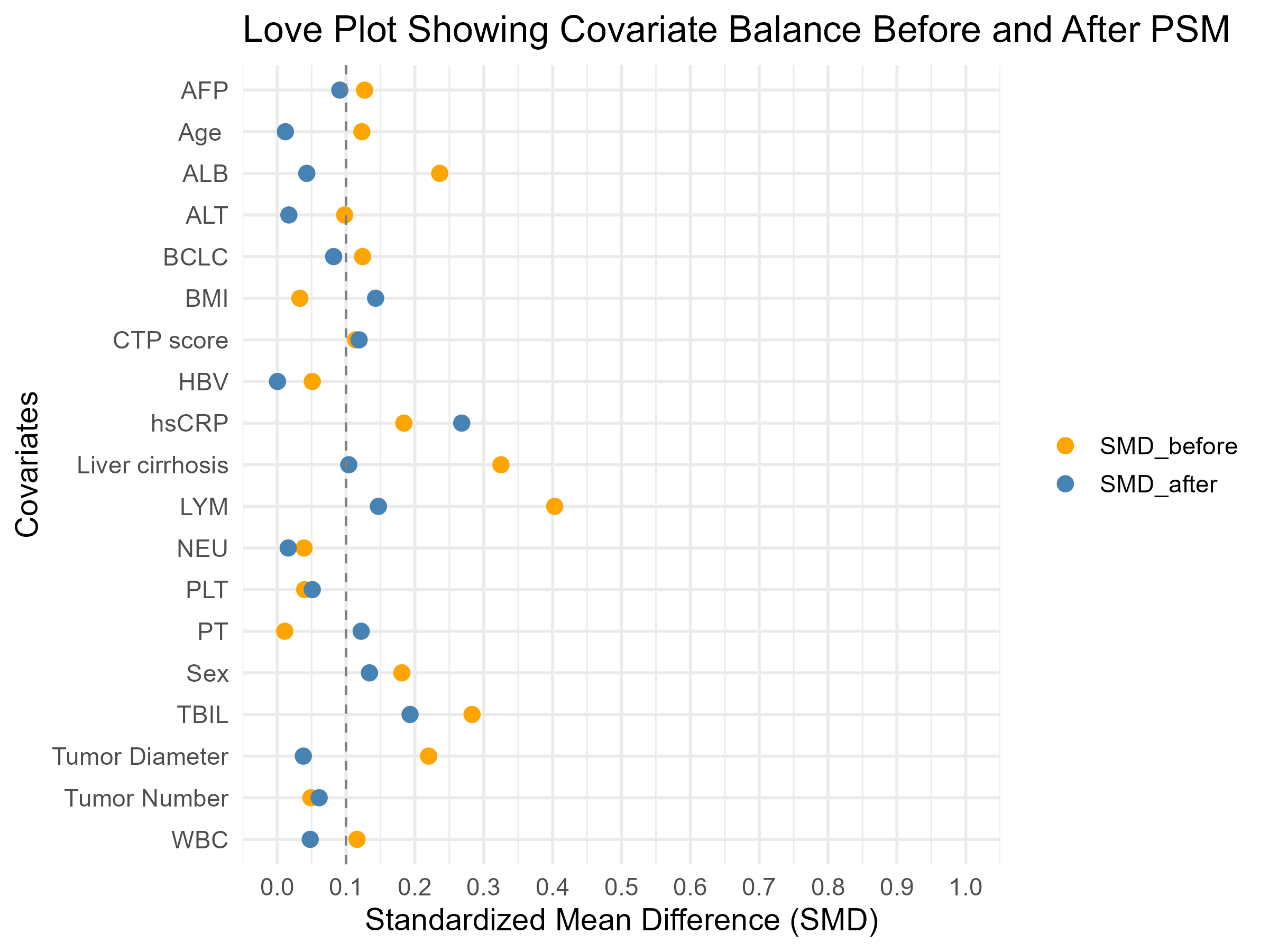
**

**Figure S1.** Love plot showing the covariate balance before and after propensity score matching (PSM). Standardized mean differences (SMDs) for each baseline variable are displayed before (orange) and after (blue) matching.

**Abbreviations:** SMD, standardized mean difference; PSM, propensity score matching; AFP, alpha-fetoprotein; ALB, albumin; ALT, alanine aminotransferase; BCLC, Barcelona Clinic Liver Cancer; BMI, body mass index; CTP, Child–Turcotte–Pugh; HBV, hepatitis B virus; hsCRP, high-sensitivity C-reactive protein; LYM, lymphocyte; NEU, neutrophil; PLT, platelet; PT, prothrombin time; TBIL, total bilirubin; WBC, white blood cell.


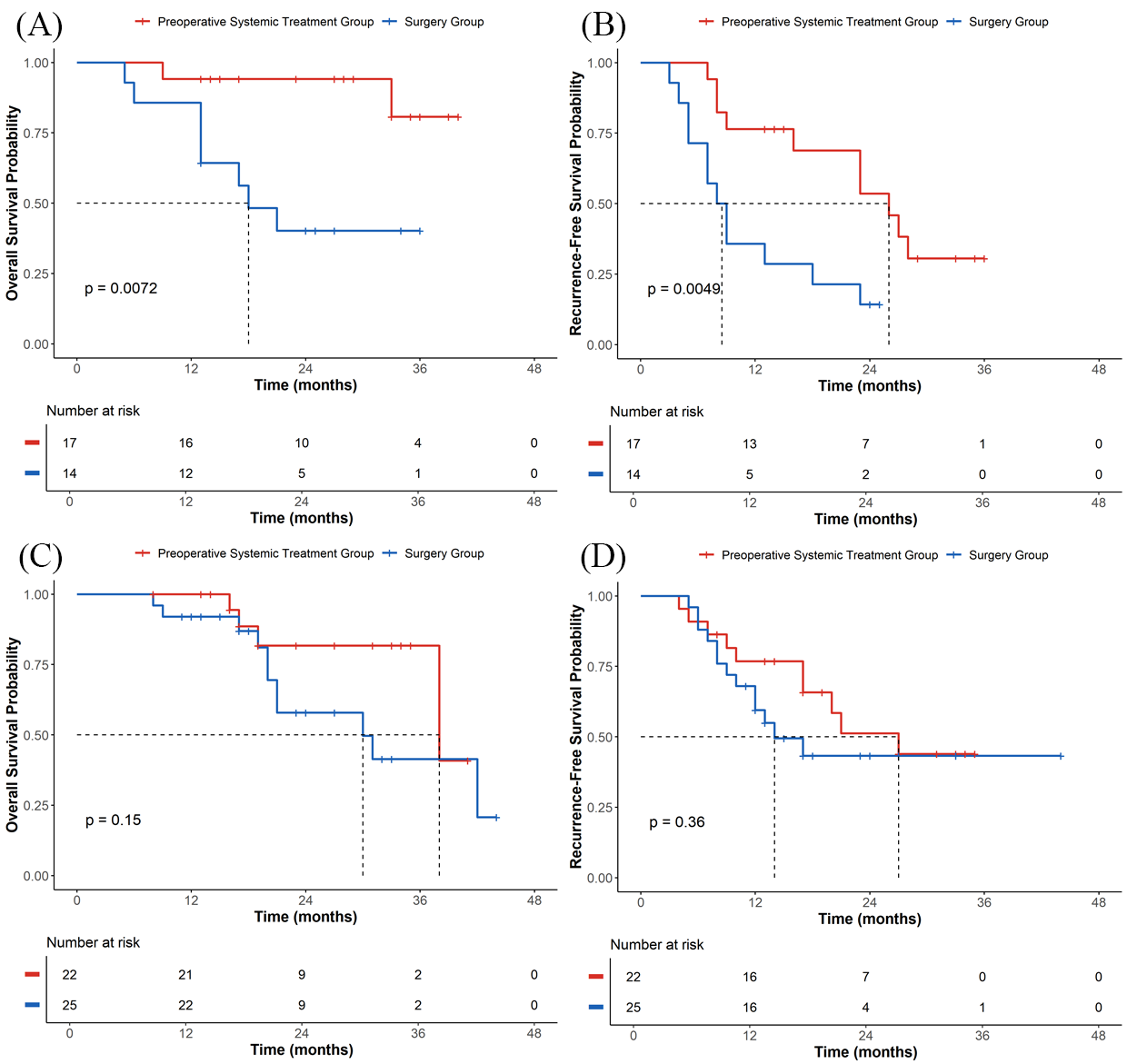


**Figure S2. Kaplan–Meier survival curves stratified by microvascular invasion (MVI) status in the post-PSM cohort.**
(A) Overall survival (OS) in patients with MVI-positive tumors.
(B) Recurrence-free survival (RFS) in patients with MVI-positive tumors.
(C) OS in patients with MVI-negative tumors.
(D) RFS in patients with MVI-negative tumors.


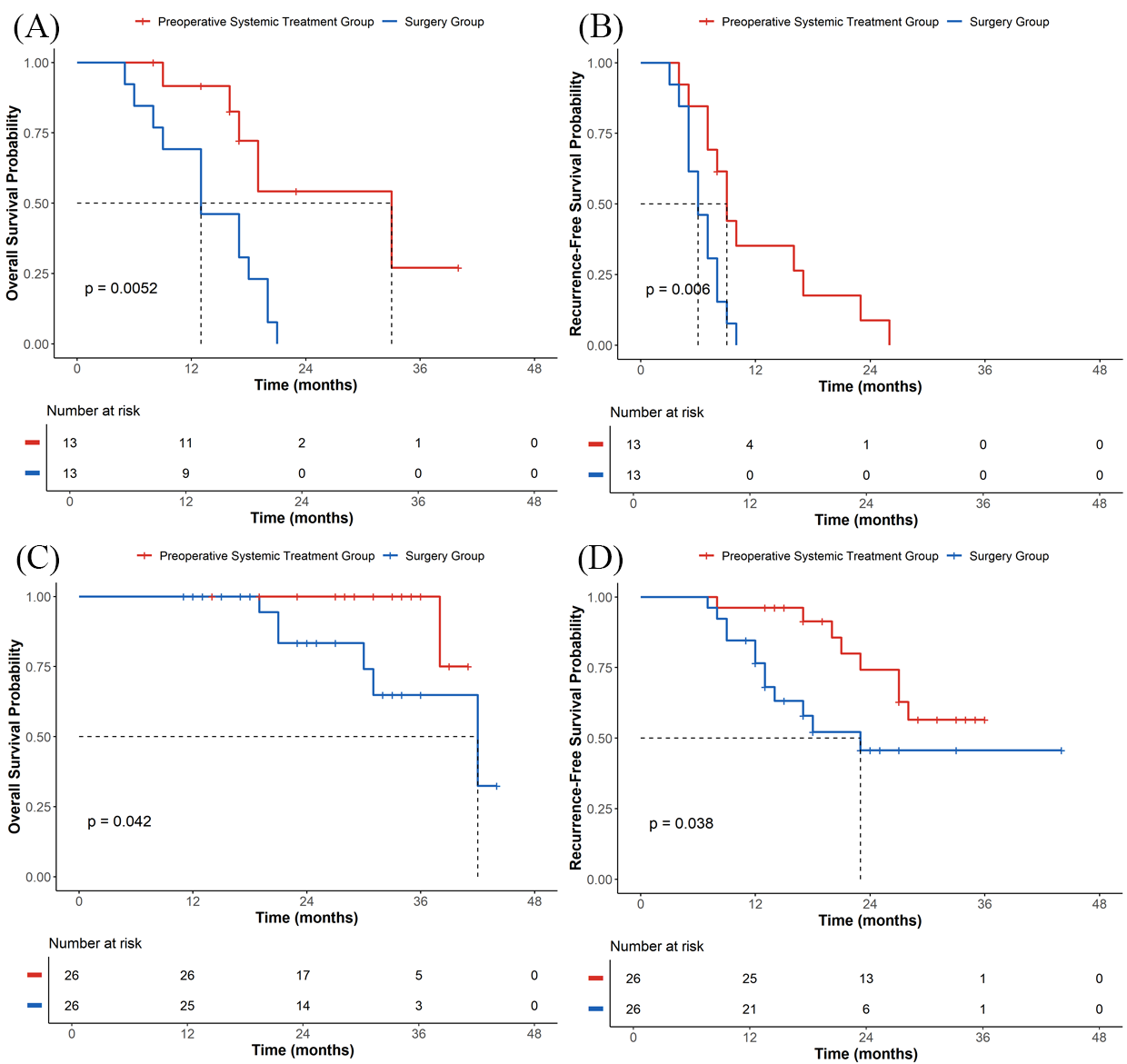


**Figure S3. Kaplan–Meier survival curves stratified by histologic differentiation in the post-PSM cohort.**
(A) Overall survival (OS) in patients with Edmondson–Steiner grade III–IV tumors.
(B) Recurrence-free survival (RFS) in patients with Edmondson–Steiner grade III–IV tumors.
(C) OS in patients with grade I–II tumors.
(D) RFS in patients with grade I–II tumors.
